# Supplementary material for: Magnetically driven capsules with multimodal response and multifunctionality for biomedical applications
Source: Nat Commun. 2024 Feb 29;15:1839. doi: 10.1038/s41467-024-46046-9 (PMC10904804; doi:10.1038/s41467-024-46046-9)
Supplement: Supplementary file 1 — Supplementary Information [file 41467_2024_46046_MOESM1_ESM.docx]

Supplementary Materials

**Magnetically Driven Capsules with Multimodal Response and Multifunctionality for Biomedical Applications**

*Yuxuan Sun^1,2^†, Wang Zhang^1,2^†, Junnan Gu^3^, Liangyu Xia^1,2^, Yinghao Cao^4^, Xinhui Zhu^1,2^, Hao Wen^1,2^, Shaowei Ouyang^1,2^, Ruiqi Liu^1,2^, Jialong Li^1,2^, Zhenxing Jiang^3^, Denglong Cheng^3^, Yiliang Lv^1,2^, Xiaotao Han^1,2^, Wu Qiu^5^, Kailin Cai^3^, Enmin Song^6^, Quanliang Cao^1,2*^, Liang Li^1,2*^*

*Corresponding authors. Email: quanliangcao@hust.edu.cn (Q.C.); liangli44@hust.edu.cn (L.L.)

**The PDF file includes:**

Note S1. Multi-functional capsules.

Note S2. Deformation analysis of magnetic soft valve.

Note S3. Fluid-solid coupling analysis.

Note S4. Locomotion mechanism.

Note S5. Description of the process of MagCap movement in the anatomical model.

Note S6. Calibration experiments with labeled drugs.

Note S7. Mechanical testing.

Note S8. Silica coating of magnetic particles

Fig. S1. Dimension diagram of the MagCaps (Capsule 1-6).

Fig. S2. Supplementary data on magnetic and mechanical properties of MagCap.

Fig. S3. Opening angle of a magnetic leaf of the same magnetic field strength under the action of magnetic frames of different mass fractions.

Fig. S4. Comparison of experimental and simulated bending angles without any magnetic frame for different magnetic fields.

Fig. S5. Capsule sealing test of magnetic frames with different magnetic powder contents.

Fig. S6. Pictures of electromagnetic coils, power supplies and robotic arm.

Fig. S7. Demonstration of the simultaneous drug release process for dual-module capsules with large magnetic field **B_h2_**.

Fig. S8. The mixing process of two different colorant drugs by the threaded capsule with a medium-frequency magnetic field (10 Hz).

Fig. S9. Preparation process for the magnetic leaf and magnetic frame.

Fig. S10. Acid resistance test for each part of MagCap.

Fig. S11. Relationship between labeled drugs and absorbance.

Fig. S12. Finite element simulation model and specific structural parameters of the poly-magnetic coil.

Fig. S13. Fluid exchanging processes within a capsule under the action of an applied magnetic field.

Fig. S14. Multimodal locomotion mechanisms of capsules.

Fig. S15. Driving magnetic field waveforms of the MagCap in various cases.

Table S1. Comparison of the proposed MagCaps with the ones previously reported in the literature.

Table S2. Structural features of various capsules and their corresponding function.

Table S3. Magnetic actuation systems used in different experimental scenarios.

Table S4. Structural features of various coils.

**Supplementary Notes:**

**Note S1.** **Multi-functional capsules.**

Based on our previous work^1-4^, we were able to prepare and program magnetic soft robots within the millimeter scale, which were then integrated into MagCap, and in this paper we provide miniature capsules (referred to **cap1** in **Fig. S1**) of 6 mm diameter and 13 mm length. Theoretically, we could have provided capsules in smaller sizes, however, the precision and cost limitations of 3D printing for biocompatibility restrict us from achieving smaller sizes. Commercial applications can overcome this limitation through methods such as mold opening.

To further investigate the dynamic characteristics of MagCap, we have enlarged the capsule by 1.5 times to obtain larger capsule (referred to **cap2** in **Fig. S1**) with excellent observational capabilities, owing to their transparent outer shell, we can easily observe the state of the magnetic leaf and the liquid inside the capsule. On the basis, we further expand the diversity of capsules by proposing dual-module capsule (referred to **cap3** in **Fig. S1**), threaded capsule (referred to **cap4** in **Fig. S1**), light-assisted therapy capsule (referred to **cap5** in **Fig. S1**), and thermal-assisted therapy capsule (referred to **cap6** in **Fig. S1**). These morphologically and structurally different magnetically-controlled capsules can be used for combined drug delivery of multiple drugs, small intestinal villi/mucus removal, and wireless photothermal therapy, etc., respectively. The specific dimensions of the various capsules are shown in the **Fig. S1**.

**Note S2.** **Deformation analysis of magnetic soft valve**

In order to qualitatively discuss the dynamic response characteristics of magnetic soft valves under an applied magnetic field, we utilized the generalized neo-Hookean model to predict the deformation behavior of magnetic soft leaves. According to Zhao et al.^5^, the potential energy per unit volume *W* of the magnetic leaf during deformation can be divided into elastic potential energy ($W^{\text{elastic}}$) and magnetic potential energy ($W^{magneitc}$) when the actuating magnetic field was applied.

|  | $W^{\text{elastic }}=\frac{G}{2}\left(J^{-2/3}I_{1}-3\right)+\frac{K}{2}(J-1)^{2}$ | (S1) |
| --- | --- | --- |
|  | $W^{\text{magnetic }}=-J[(\mathbf{FM}/J)\cdot\mathbf{B}_{\text{a}}]=-\mathbf{FM}\cdot\mathbf{B}_{\text{a}}$ | (S2) |

where $G$ and $K$are the soft material’s shear modulus and bulk modulus, respectively, $\mathbf{F}$ is the deformation gradient tensor, $J$ is the deformation Jacobian of $\mathbf{F}$, and $I_{1}=\text{tr}(\mathbf{F}^{\text{T}}\mathbf{F})$, $\mathbf{M}$ is the magnetization. Based on work-conjugation, the Cauchy stress can be calculated by

|  | $\mathbf{\sigma}=\frac{1}{J}\frac{\partial W}{\partial F}F^{T}=GJ^{-5/3}\left(\mathbf{FF}^{\text{T}}-\frac{I_{1}}{3}\mathbf{I}\right)+K\left(J-1\right)\mathbf{I}-\frac{1}{J}\mathbf{B}\otimes\mathbf{FM}$ | (S3) |
| --- | --- | --- |

In all the simulations displayed in **Fig. S4**, the deformed structures in response to the actuation magnetic fields were simulated by a user-defined element (UEL) subroutine with the commercial finite-element analysis software ABAQUS. Magnetization and Poisson’s ratio of the magnetic leaf a were set to 80.5 kA/m, and 0.495, respectively. In addition, the shear modulus *G* and the bulk modulus *K* are set to 80 kPa and 1000 *G* (the large bulk modulus was chosen to approximate incompressibility), respectively.

**Note S3. Fluid-solid coupling analysis**

In Fig. 3d of the manuscript, the experiment was conducted by placing the MagCaps within a receptacle filled with liquid, and subsequently subjecting them to a high-frequency (10-60 Hz) magnetic field. Throughout the experiment, the magnetic leaf within the MagCaps underwent periodic bending, showcasing a characteristic fluid-solid coupling phenomenon. We contend that simulating this phenomenon will aid in comprehending the functional principles of the MagCaps.

In this study, finite element software COMSOL was utilized for analysis, and a 2D model was developed. Despite the distance between the 2D model simulation and the actual experiment, it remains valuable for qualitatively analyzing changes in the flow field. In the simulation, the magnetic valve is divided into *i* equivalent elements, and the distributed magnet torque is substituted by the cumulative magnetic torque, achieved through two tangential surface forces (in N·m^-2^) relative to the boundary frame^6^.

|  | $F_{i}=M_{x}^{i}B_{y}-M_{y}^{i}B_{x}$ | (S4) |
| --- | --- | --- |

where $M_{x}^{i}$ and $M_{y}^{i}$ represent the average magnetization of the element in the x_g-_ and y_g-_axes, and *B_x_* and *B_y_* are the components of the magnetic flux density magnitude B in the x_g-_ and y_g-_directions, respectively.

The flow field surrounding the magnetic leaf is unsteady and nonuniform. Therefore, we solve the complete Navier-Stokes equation involving the inertial, convection, pressure, and diffusion terms. The fluid is assumed to be Newtonian and incompressible. Considering the mass and linear momentum balance, the physical behavior of the fluid is captured through the following equations:

|  | $\nabla\cdot\mathbf{u}=0$ | (S5) |
| --- | --- | --- |
|  |  | (S6) |

Here, *p* represents the scalar pressure field, ***D*** denotes the rate of deformation tensor, and ***u*** is the velocity field. *μ* and *ρ_f_* represent the fluid viscosity and density, respectively.

Utilizing the aforementioned model, we can simulate the fluid effects arising from the periodic deflection of the magnetic leaf induced by an alternating magnetic field (**Fig. S13**). From the onset to the quarter cycle (0-0.25 T), the magnetic leaf deflects upwards, leading to an increase in pressure within the capsule cavity. The liquid inside the capsule then extends in a counterclockwise flow, with a portion of the internal liquid flowing out through the channel. As the deflection angle of the magnetic leaf increases (0.5 T), liquid outside flows into the capsule along the channel's side. Simultaneously, a vortex ring forms beneath the magnetic leaf, initiating a rapid mixing process between the internal chamber liquid and the inflowing external liquid. In the latter half of the cycle (0.5-1 T), the magnetic leaf gradually approaches closure. During this phase, a portion of the mixed liquid enters the interior of the capsule, while another portion exits the capsule. For instance, considering a frequency of 30 Hz, the MagCap undergoes 30 analogous cycles within a second under the influence of a high-frequency magnetic field. This phenomenon establishes the foundation for the release and sampling within the MagCaps.

**Note S4. Locomotion mechanism**

The movement of the MagCaps along the S-bend track (Fig. 4b) can be considered planar motion, and it can be roughly categorized into two modes (**Fig. S14**): rolling and turning. The axial magnetization of the magnetic frame and the radial symmetric magnetization of the magnetic leaf provide the MagCap with an overall single magnetic orientation (**M_1_**), which lays the foundation for the MagCap’s controllable mobility. In the presence of a uniform magnetic field, the MagCap will roll until the magnetization direction of the magnetic frame inside the capsule aligns with the direction of the magnetic field. In cases where the applied uniform magnetic field rotates, the capsule will simultaneously rotate and roll either forward or backward. When the capsule requires movement from Pos_1 to Pos_2 (**Fig. S14-b**), with **B_x1_** representing the initial desired magnetic field and **n** as the direction vector of the rotation axis, the required magnetic field **B_y1_**, necessary to induce the roll can be expressed as follows^7^:

|  |  | (S7) |
| --- | --- | --- |

where is a rotation matrix that rotates angle about **n** axis. When **n** is in the XOY plane and orthogonal to the moving direction of the capsule, the capsule can keep moving forward by continually updating **B_y1_**.

Hence, Based on the theoretical analysis described above and Eq. (S7), in the rolling mode depicted in **Fig. S14-b**, the magnetic torque $\mathbf{\tau}_{12}$ acting on the MagCap is expressed in terms of the driving magnetic field $\mathbf{B}_{y1}$ and the residual magnetization strength $M_{1}$, respectively, as

$\mathbf{\tau}_{12}=M_{1}\times\mathbf{B}_{y1}$ (S8)

where $\mathbf{B}_{y1}$ is the high frequency alternating magnetic field generated by an external coil, and $m_{1}$ is the residual magnetization strength retained by the magnetized NdFeB particles in the magnetic frame. For this reason, the capsule can be tumbled around the Z-axis by generating a rotating magnetic field in the XOY plane. The specific drive waveform is illustrated in **Fig. S15a**, with the signal being output from the STM32 (**Fig. S6d**) and subsequently normalized.

Furthermore, we can introduce a magnetic field along the Z-axis into the 2D magnetic field, as depicted in **Fig. S14c**. Therefore, the magnetic torque$\mathbf{\tau}_{34}$ responsible for turning the capsule to the left can be calculated using the following equation.

$\mathbf{\tau}_{34}=M_{1}\times\mathbf{B}_{z4}$ (9)

The magnetic field waveform that actuates the capsule is depicted in **Fig. S15b**. The rotation of the MagCap can be controlled by changing the direction of the rotating magnetic field. By detecting the push direction from the handle pusher, the MagCap can roll toward the 45-degree direction using the waveform in **Fig. S15c**.

**Note S5. Description of the process of MagCap movement in the anatomical model**

As shown in **Fig. 4D**, under the excitation of the permanent magnet, the MagCap can perform different motion modes, including rotation, sliding, and rolling, to adapt to different environments. For example, under the influence of magnetic torque, the capsule can sway left and right to adjust its direction and swinging across the crease under the traction of gradient forces (Stage I Swinging). When the capsule slides into a groove, it can rotate along the circumference direction by moving the permanent magnet forward, using the magnetic torque along the circumference direction to overcome the friction on the groove surface, and thus sliding along the groove (Stage II Sliding). When the MagCap encounters a narrow groove, it can use the rotational motion of the permanent magnet to put itself in a rolling mode to cross it (Stage III Rolling). Experimental results demonstrate that even when facing challenges such as slippery surfaces and frictional resistance from the stomach wall model, the MagCap's motion remains controllable and adaptive in different environments. Once it reaches the designated drug release area, an oscillating magnetic field (30 mT, 30 Hz) is applied, causing an unbalance in the magnetic attraction between the capsule's magnetic leaf and the magnetic frame. As a result, the magnetic leaf undergoes bending deformation, leading to the leakage of the drug (Ponceau S) into the vicinity of the target area. The drug release rate gradually decreases as the concentration of the drug solution inside the capsule decreases. The drug release area covers approximately ~402 mm^2^. Therefore, the proposed MagCap can achieve precisely targeted drug delivery under the influence of an oscillating magnetic field.

**Note S6. Calibration experiments with labeled drugs**

In the experiments, we utilized organic dyes, Ponceau S (C_18_H_14_N_2_Na_2_O_7_S_2_, Aladdin Chemical Reagent Co., Ltd.) and Methyl blue (C_37_H_27_N_3_Na_2_O_9_S_3_, Aladdin Chemical Reagent Co., Ltd.) as substitutes for real drugs. For the sealing-ability test, we used Ponceau S as the labeling drug. Before the experiment, we first measured the absorbance of different concentrations of Ponceau S solution using a UV-Vis spectrophotometer (UV-2600i, SHIMADZU Co., Ltd.) and plotted the standard curve of Ponceau S concentration versus absorbance. The specific steps of the experiment are as follows: we weighed 40, 80, 120, 160, and 200 mg of Ponceau S dye separately, completely dissolved them in ultrapure water, and made up to 40 mL in volumetric flasks, resulting in standard solutions with concentrations of 1, 2, 3, 4, and 5 mg/mL, respectively. Then, the different Ponceau S standard solutions with ultrapure water were diluted to prepare solutions with concentrations of 7.5, 15, 22.5, 30, and 37.5 mg/L. We measured their absorbance using a UV-vis spectrophotometer, and the results are shown in **Fig. S11a**. Next, we selected the absorbance at 518 nm of the Ponceau S dye solution as the vertical axis and its concentration as the horizontal axis performed a curve-fitting on the data, and obtained the Ponceau S concentration-absorbance standard curve was pictured in **Fig. S11b**. From the graph, it can be observed that the correlation coefficient is as high as 0.99964, indicating a linear relationship between Ponceau S concentration and absorbance. Following a procedure similar to the Ponceau S concentration-absorbance standard curve, the correlation coefficient of the Methyl blue concentration-absorbance standard curve is 0.99536, as can be seen in **Fig. S11c-d**, which represents a good linear fit.

**Note S7. Mechanical testing.**

Test pieces of the same material as the MagCap’s leaf (before and after silica coating) were used for the tensile experiments (dumbbell-like structure of type 2 in GB/T 528-2009, specimen dimensional parameters are shown in **Fig. S2d**, with an original length of 20 ± 0.5 mm). Nominal stress-strain curves (**Fig. S2c**) were plotted for both materials and the specimens were tested on a mechanical testing machine (C43.504, MTS Criterion, USA) at a stretching rate of 100 mm/min.

**Note S8. Silica coating of magnetic particles**

To improve the biocompatibility of the magnetic particles and avoid corrosion by acidic liquids *in vivo*, the hydrolysis condensation reaction of tetraethyl silicate was used to achieve the silica shell coating on the surface of the magnetic particles. The specific steps were as follows: firstly, 32 g of NdFeB particles were added in 800 ml of anhydrous ethanol (Aladdin Chemical Reagent Co., Ltd.) and an electric stirrer was set to stir vigorously at 900 r/min to avoid particle settling. Next, 48 ml of ammonia at a concentration of 28% (Aladdin Chemical Reagent Co., Ltd.) was added slowly using a dispensing funnel, and then 2 ml of tetraethyl silicate (Aladdin Chemical Reagent Co., Ltd.) was added to the mixture rapidly using a pipette, taking care to avoid the tetraethyl silicate touching the inner wall of the reaction vessel. The speed of the electric stirrer was increased to 1000 r/min and the mixture was allowed to react at room temperature for 10 h. After the reaction was completed, the mixture was washed with deionized water three times, and then with anhydrous ethanol three times to fully remove the soluble and insoluble impurities in the mixture. To fully separate the magnetic particles from the ethanol, and at the same time to avoid the influence of the external magnetic field on the magnetic properties of the magnetic particles, the heating and drying method was used instead of the magnetic separation method. The mixture was put into a drying oven and baked at 80°C for 6 h to evaporate the ethanol, and the NdFeB particles with silica shells were obtained. Finally, the particles were poured into an onyx mortar for thorough grinding, and the agglomerates formed by drying were ground to obtain a fine powder.

**Supplementary Figures:**

**
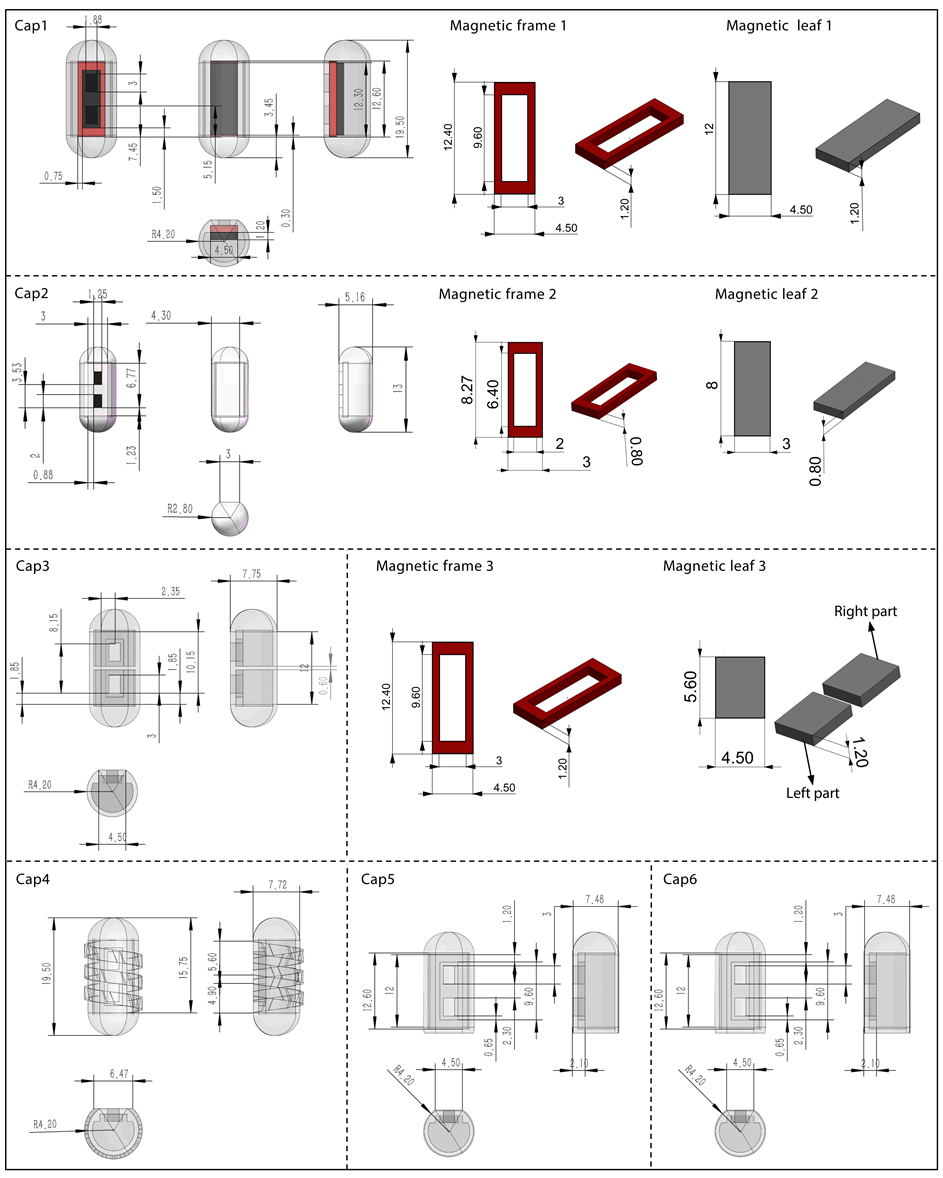
**

**Fig. S1. Dimension diagram of the MagCaps (Capsule 1-6).** All units are in millimeters.


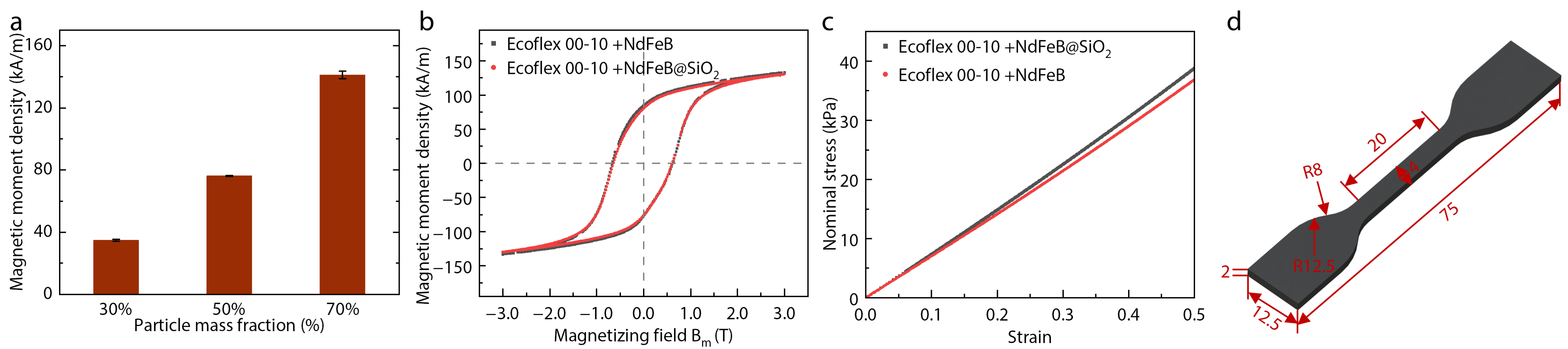


**Fig. S2. Supplementary data on magnetic and mechanical properties of MagCap** (a) Magnetization strength of magnetic frames with different mass fractions (n=3, data are presented as mean values +/- SD). Source data are provided as a Source Data file. (b) Hysteresis loop diagrams before and after coating silicon. Source data are provided as a Source Data file. (c) Nominal stress-tension curves before and after coating silicon. Source data are provided as a Source Data file. (d) Specimen dimensional parameters.


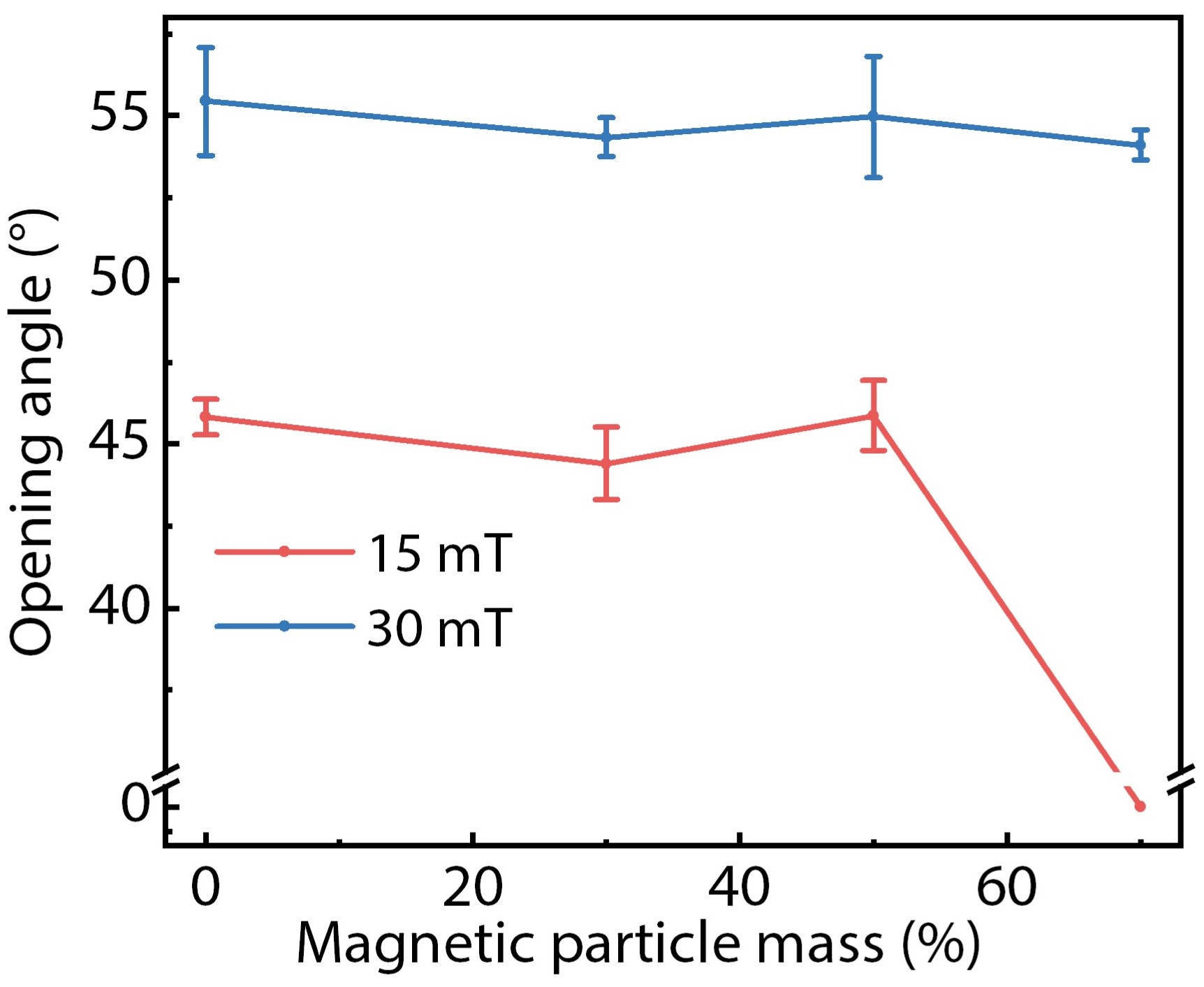


**Fig. S3. Opening angle of a magnetic leaf of the same magnetic field strength under the action of magnetic frames of different mass fractions (n=3, data are presented as mean values +/- SD). Source data are provided as a Source Data file.**


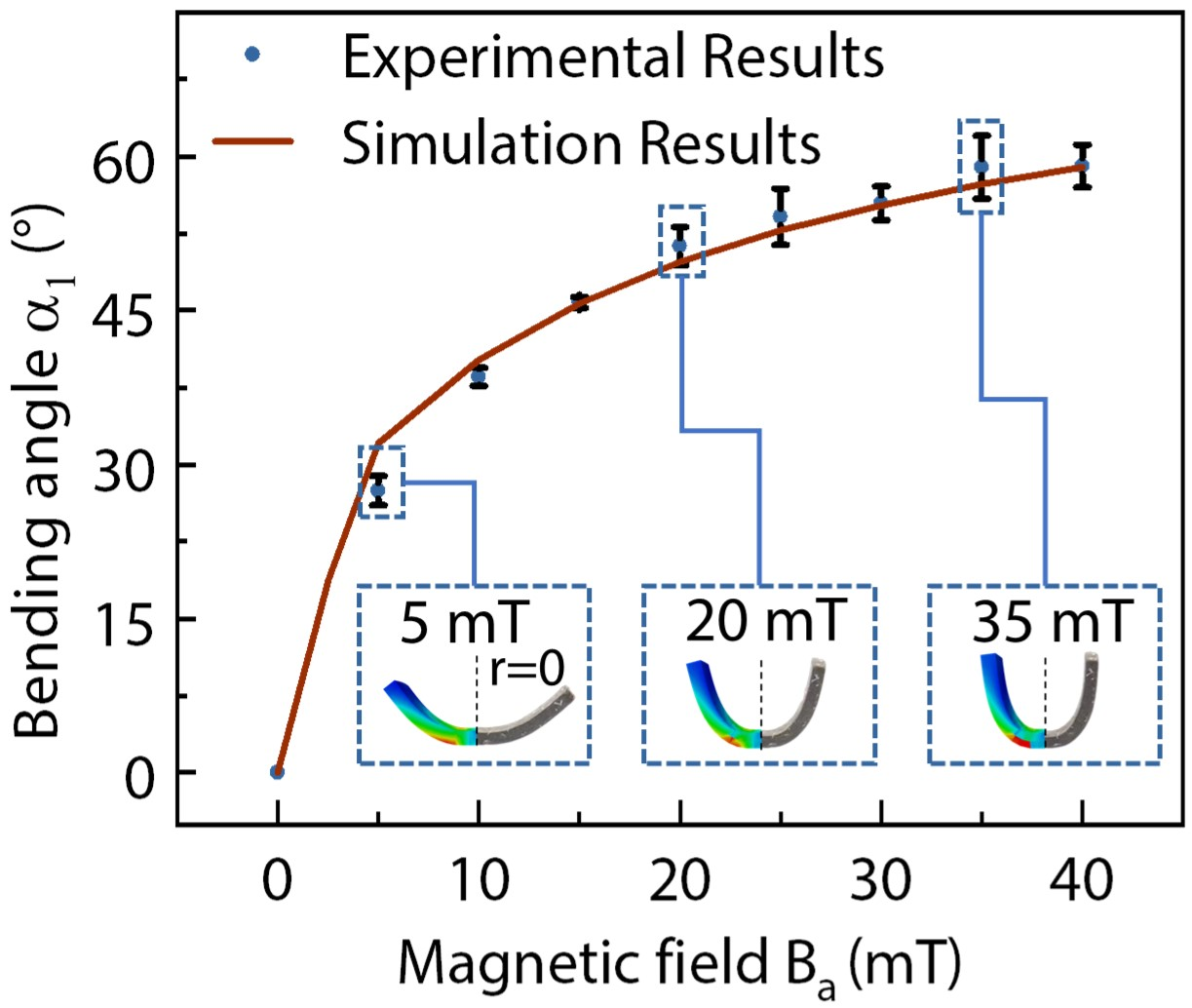


**Fig. S4. Comparison of experimental and simulated bending angles without any magnetic frame for different magnetic fields (n=3, data are presented as mean values +/- SD). Source data are provided as a Source Data file.**


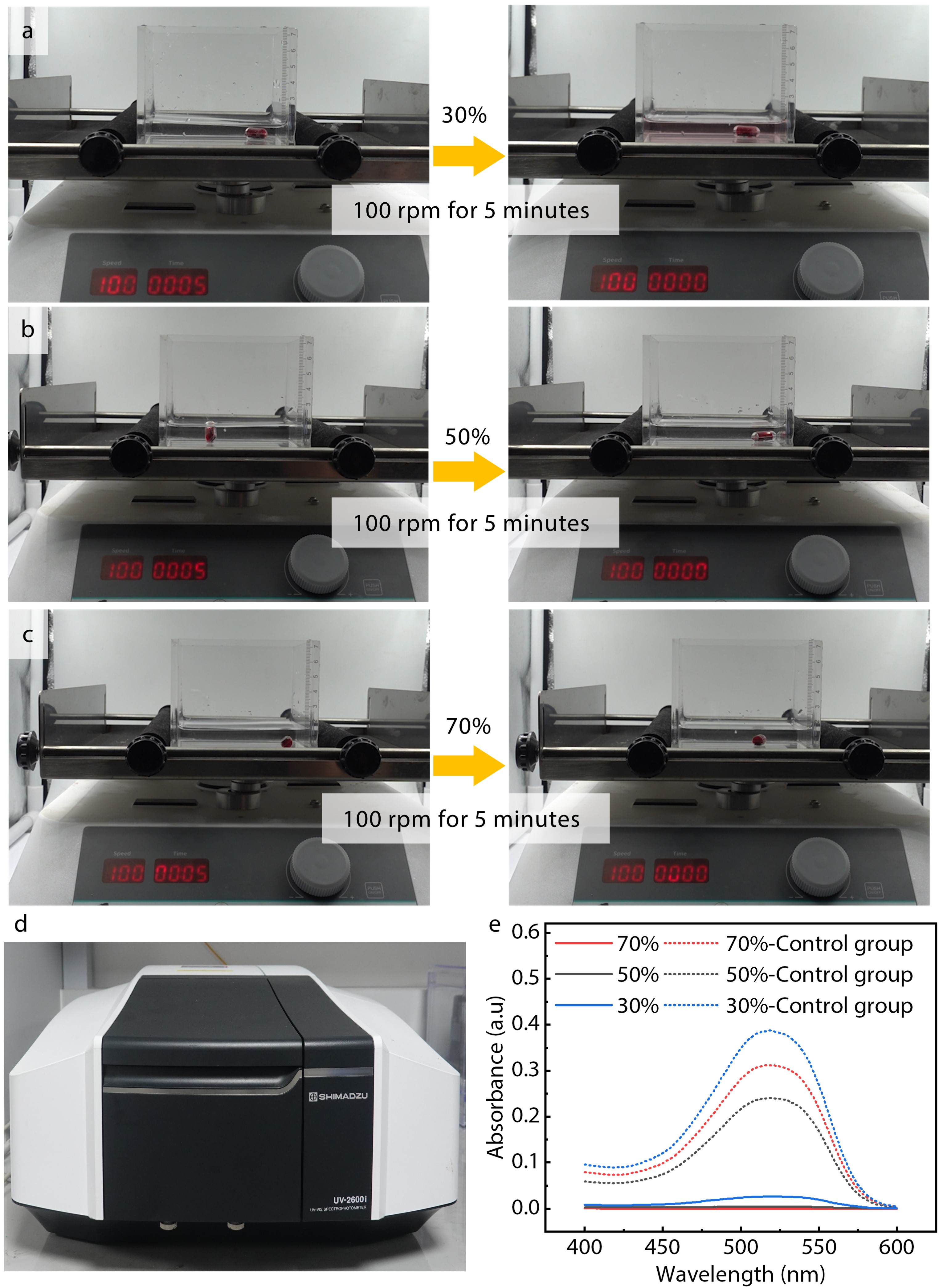


**Fig. S5. Capsule sealing test of magnetic frames with different magnetic powder contents.** (a-c) The mass fractions of magnetic locks were 30%, 50% and 70, respectively (d) Photograph of UV spectrometer, (e) Percentage of drug leakage from capsules shaken for 5 minutes with different magnetic powder contents of the magnetic frame (n=3, data are presented as mean values +/- SD). Source data are provided as a Source Data file.


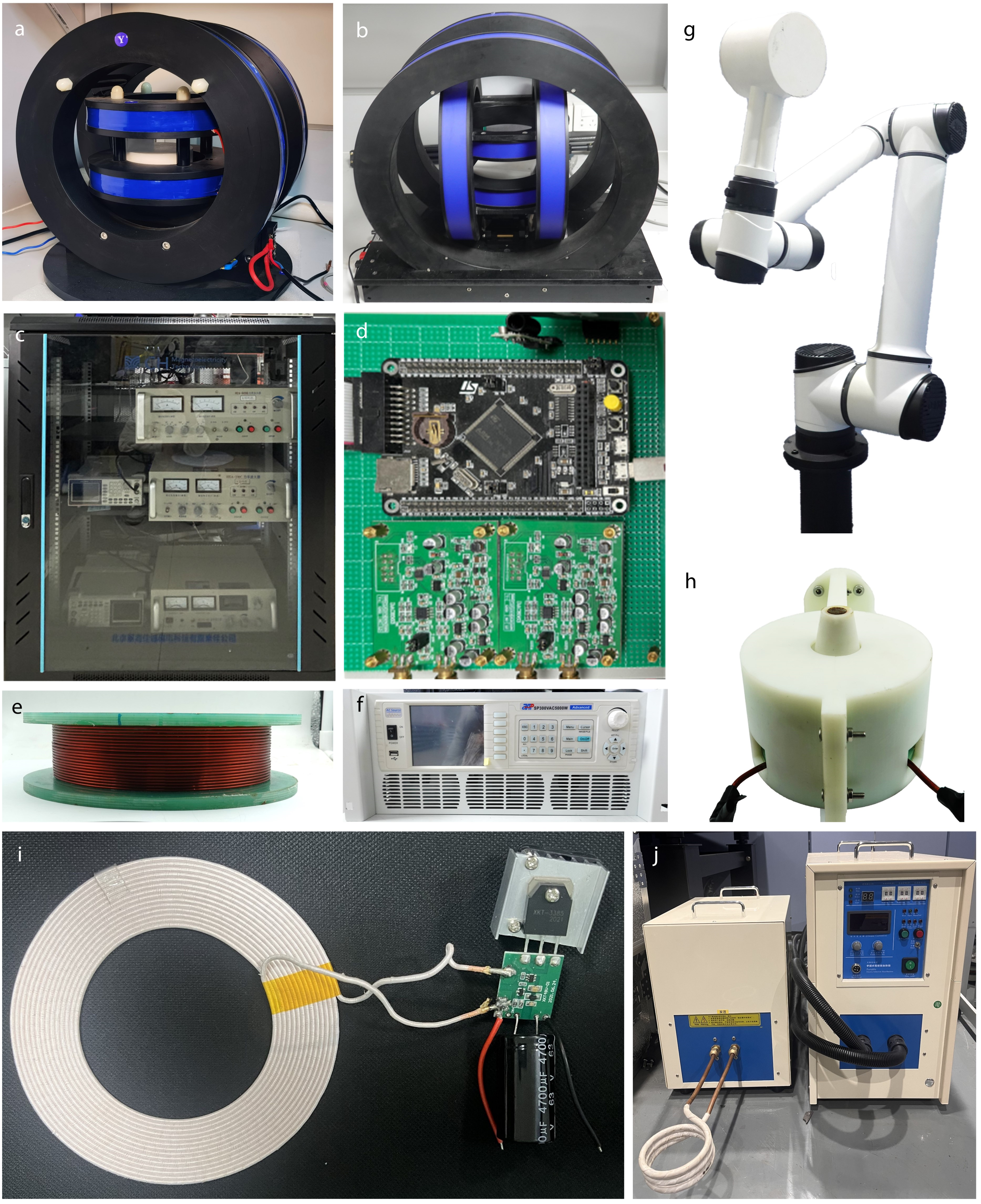


**Fig. S6.** **Pictures of electromagnetic coils, power supplies and robotic arm.** (a) Two-dimensional Helmholtz coils, (b) Three-dimensional Helmholtz coils, (c) Power amplifier, (d) STM32 control board, (e) Electromagnetic coil for drug release, (f) AC Power supply, (g) 6-degree-of-freedom robotic arm, (h) Poly-magnetic coil, (i) Wireless powered transmitter Coil, (j) Wireless heating coils.

**
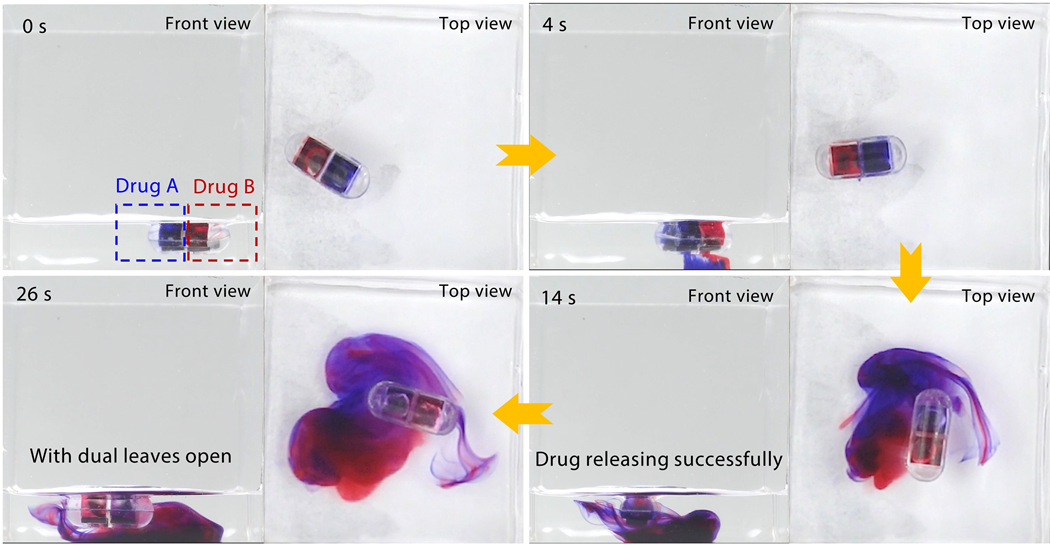
**

**Fig. S7.** **Demonstration of the simultaneous drug release process for dual-module capsule with large magnetic field B_h2_.**


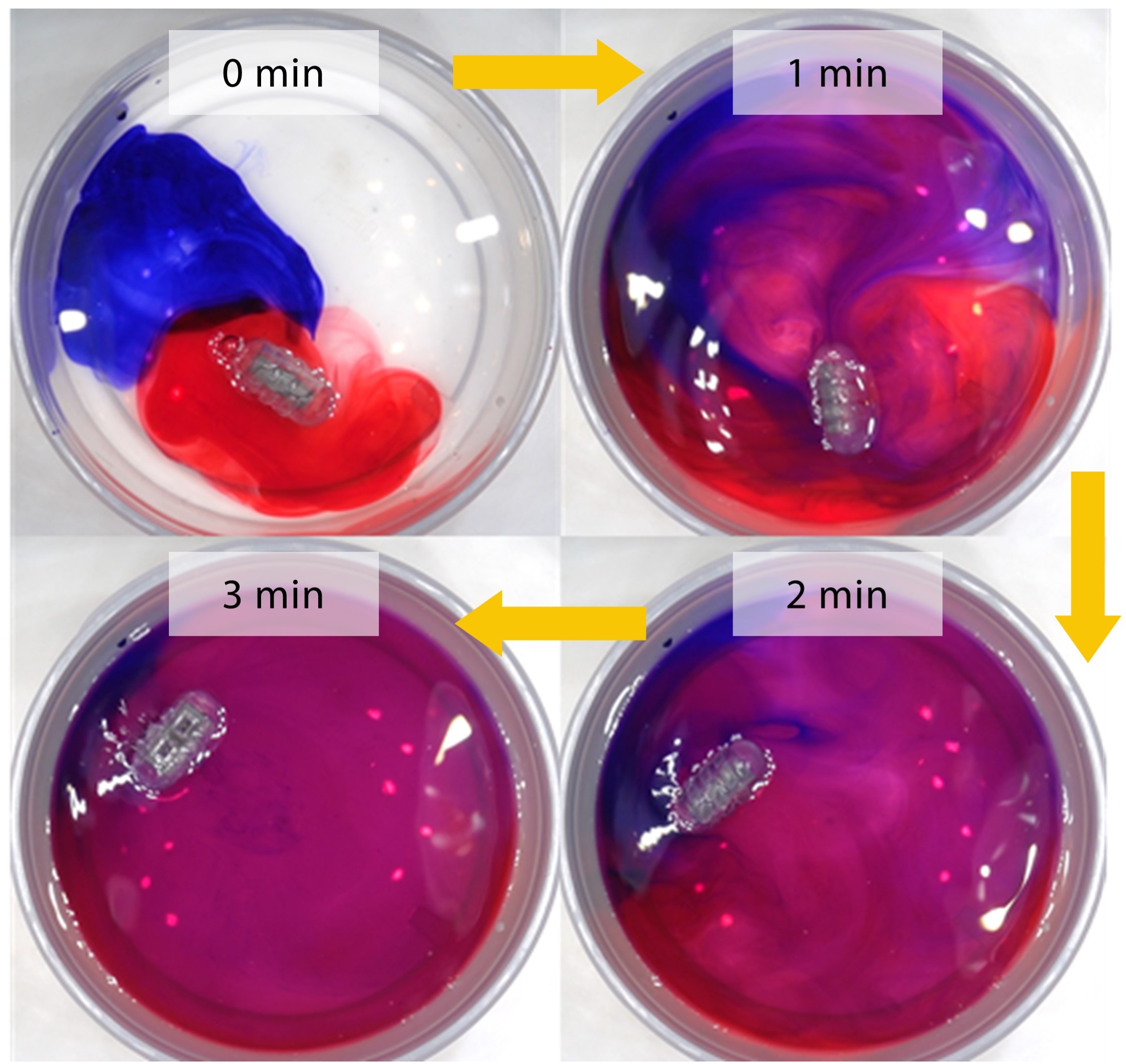


**Fig. S8. The mixing process of two different colorant drugs by the threaded capsule with a medium-frequency magnetic field (10 Hz).**


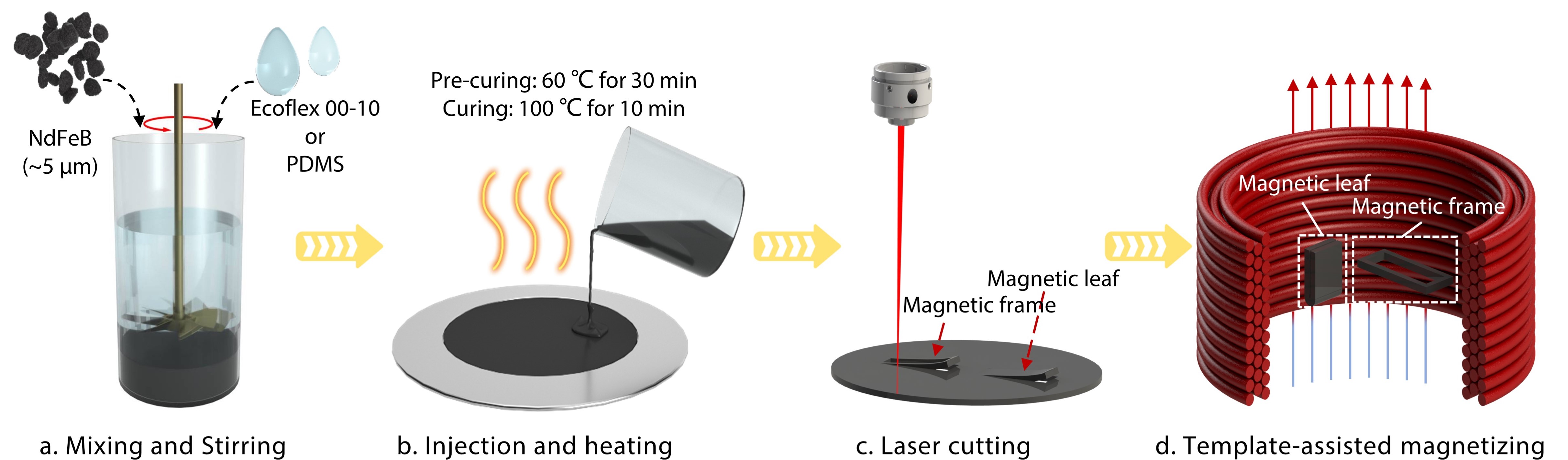


**Fig. S9. Preparation process for the magnetic leaf and magnetic frame.** (a) Mixing and stirring. (b) Injection and heating. (c) Laser cutting. (d) Template-aided magnetizing.


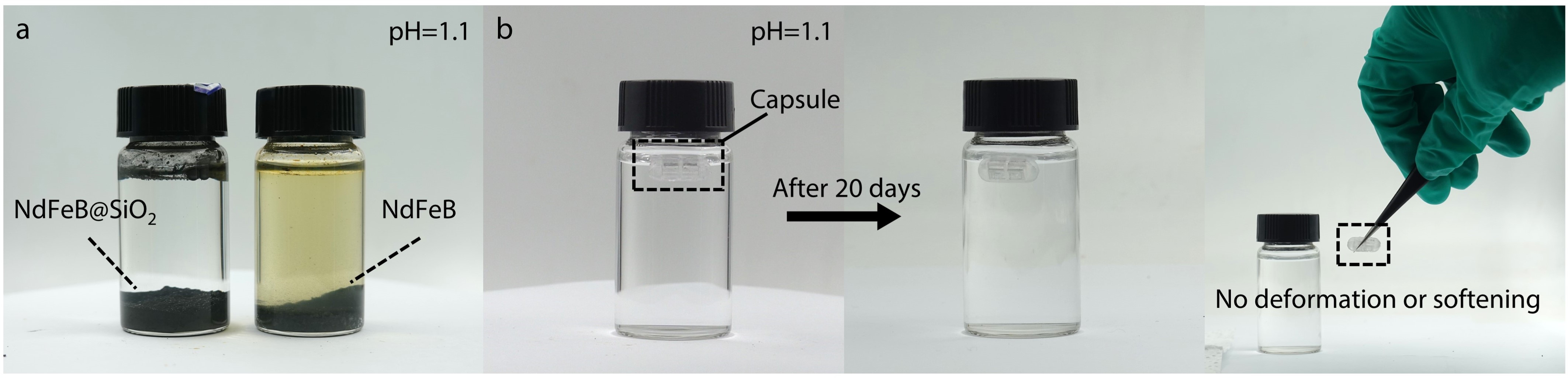


**Fig. S10. Acid resistance test for each part of MagCap.** (a) Before and after NdFeB-coated silicon. (b) MED610 shell.


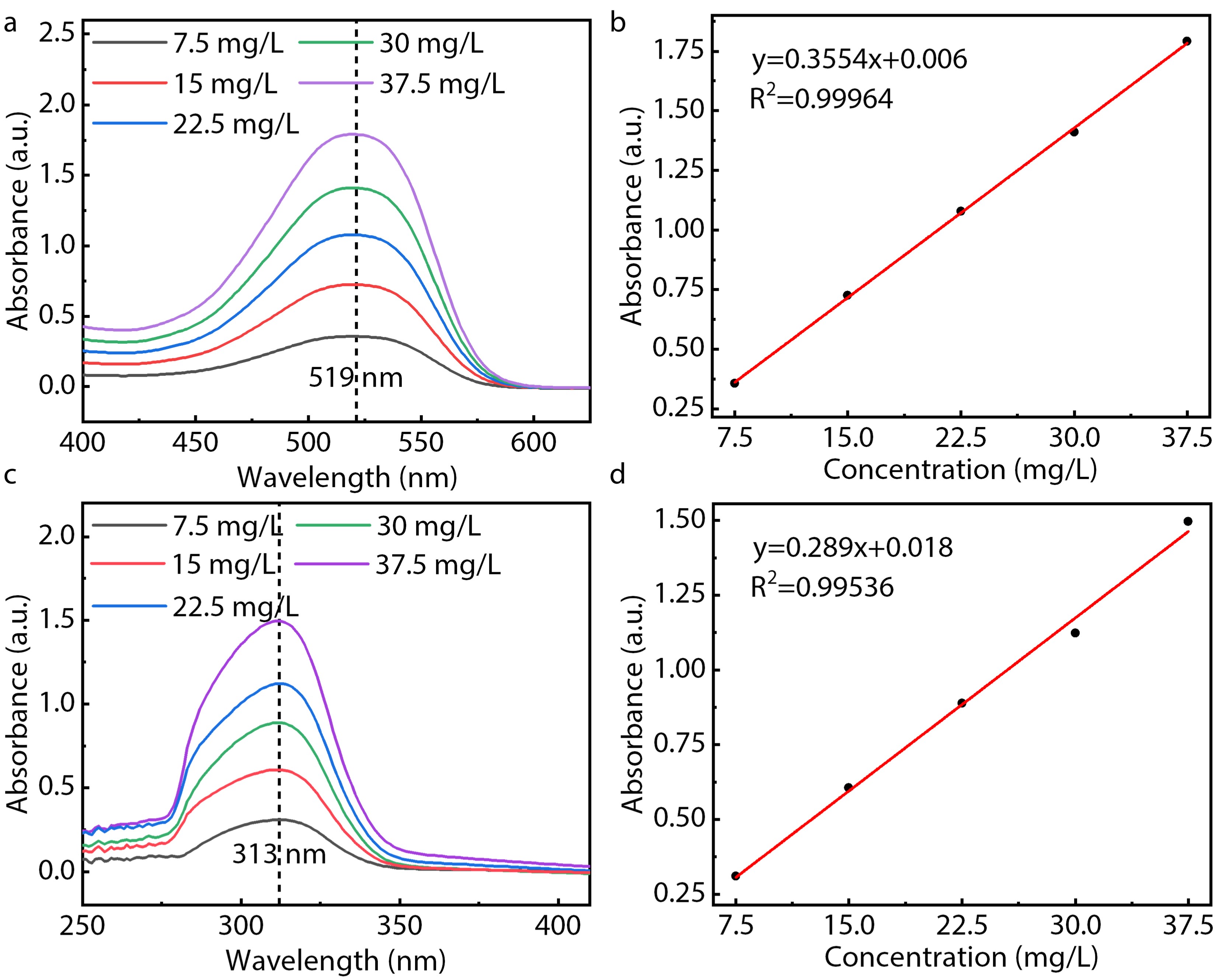


**Fig. S11. Relationship between labeled drugs and absorbance.** (a) Absorbance of different concentrations of Ponceau S solution. (b) Concentration-absorbance standard curve of Ponceau S solution. (c) Absorbance of different concentrations of Methyl blue solution. (d) Concentration-absorbance standard curve of Methyl blue solution (n=3, data are presented as mean values +/- SD). Source data are provided as a Source Data file.


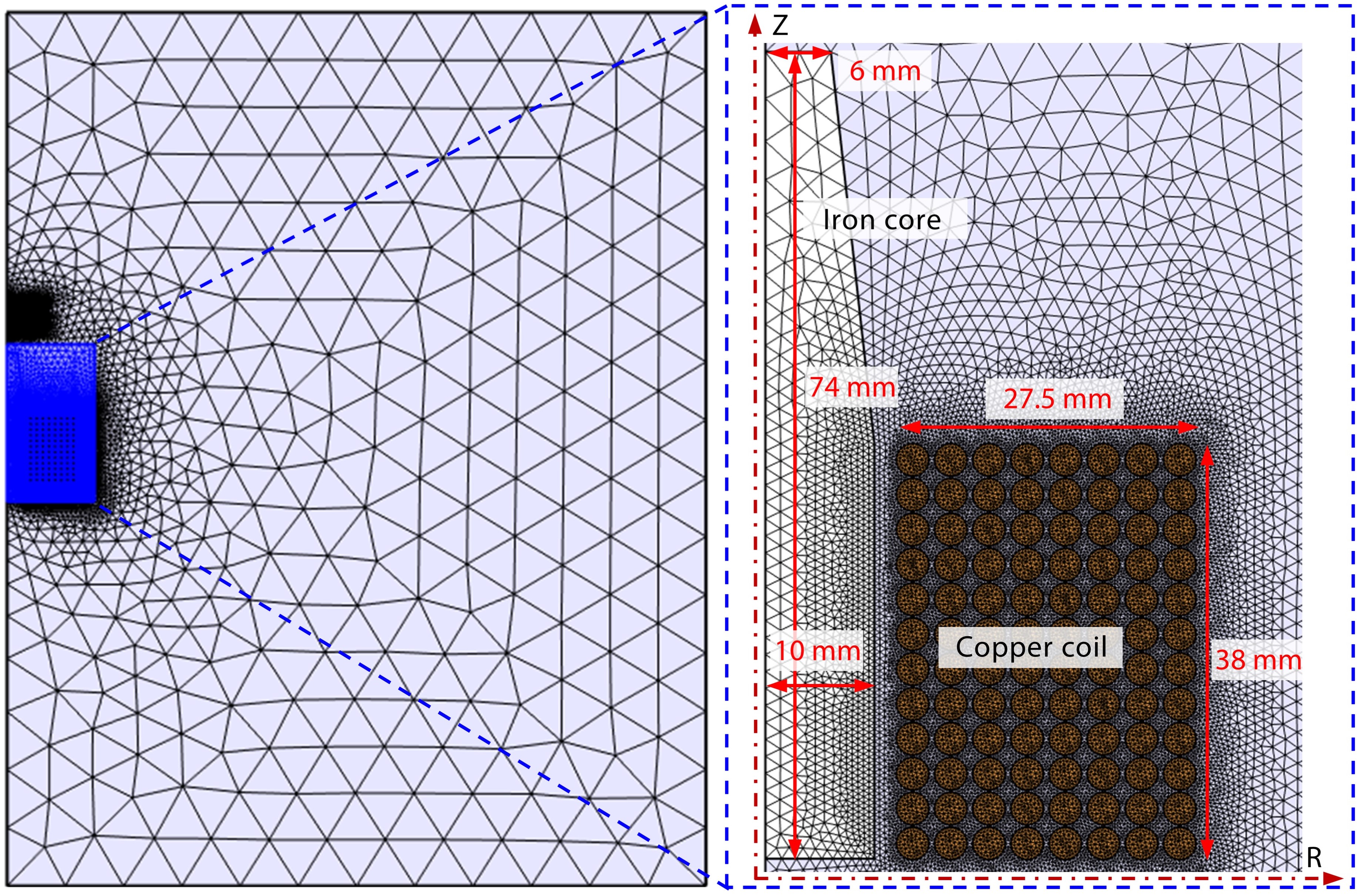


**Fig. S12.** **Finite element simulation model and specific structural parameters of the poly-magnetic coil.**


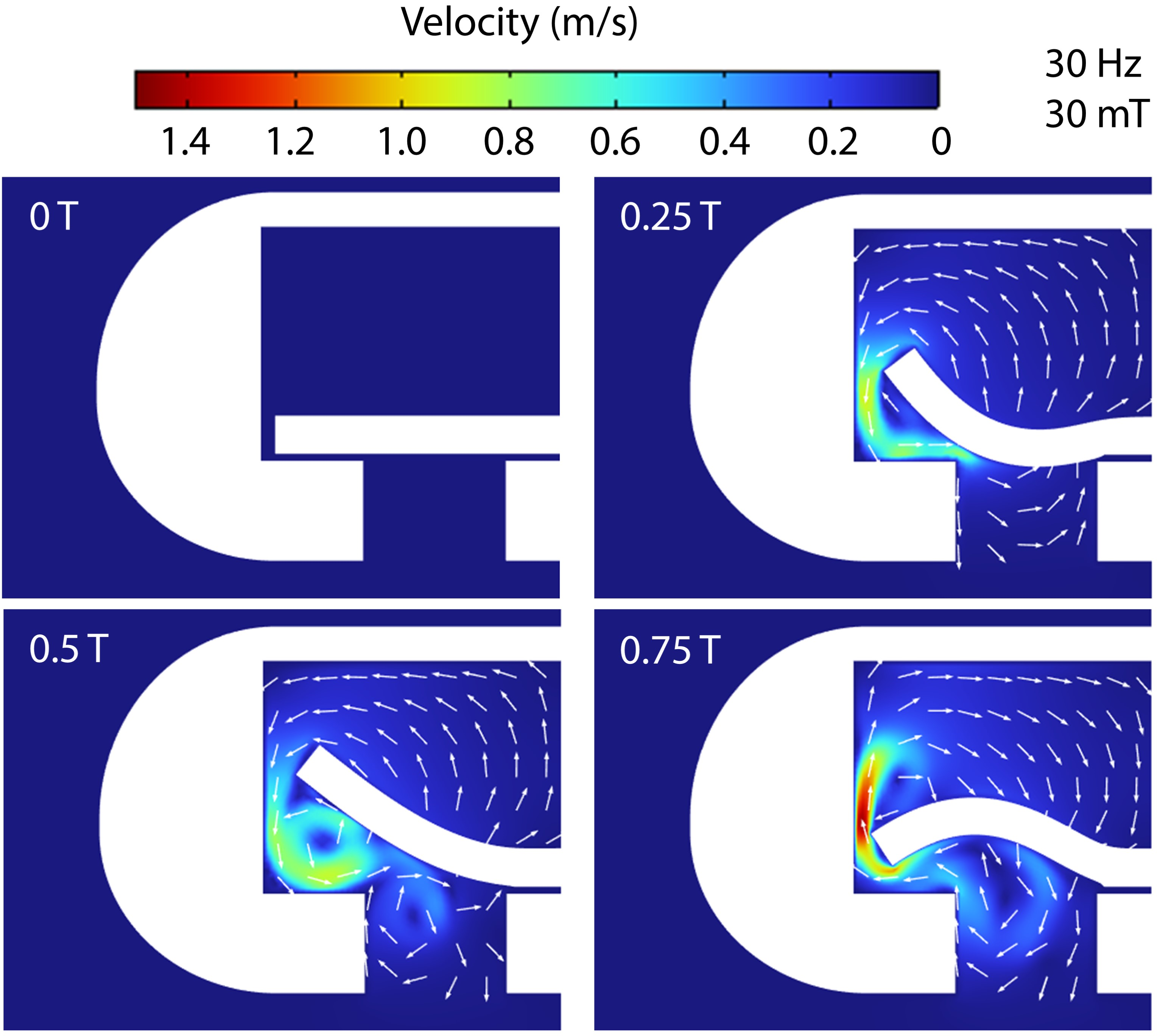


**Fig. S13. Fluid exchanging processes within a capsule under the action of an applied magnetic field.**


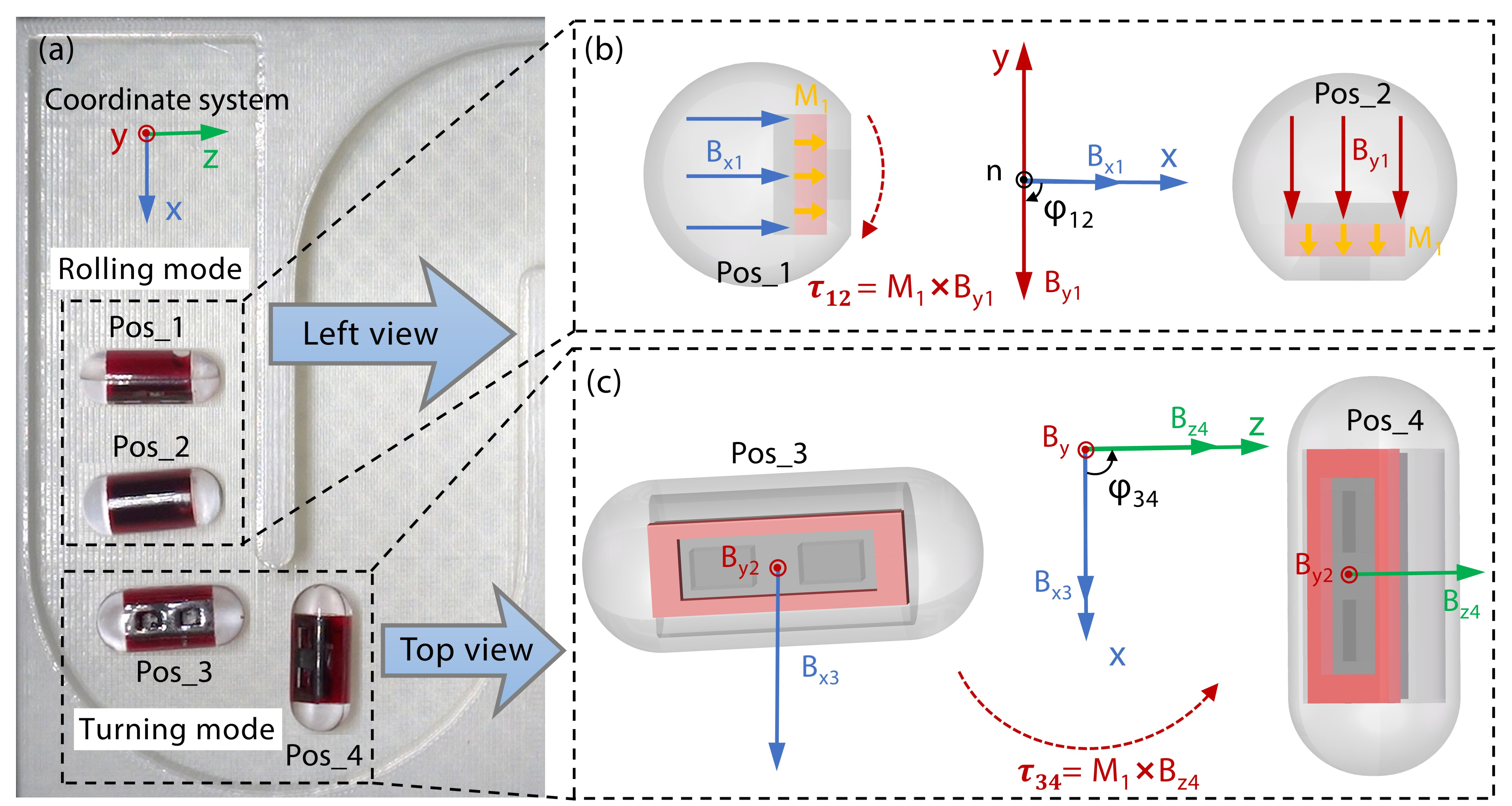


**Fig. S14. Multimodal locomotion mechanisms of capsules.** (a) Illustration of two typical locomotion modes of Magcaps. (b) Left view of the rolling mode. (c) Top view of the turning mode.

**
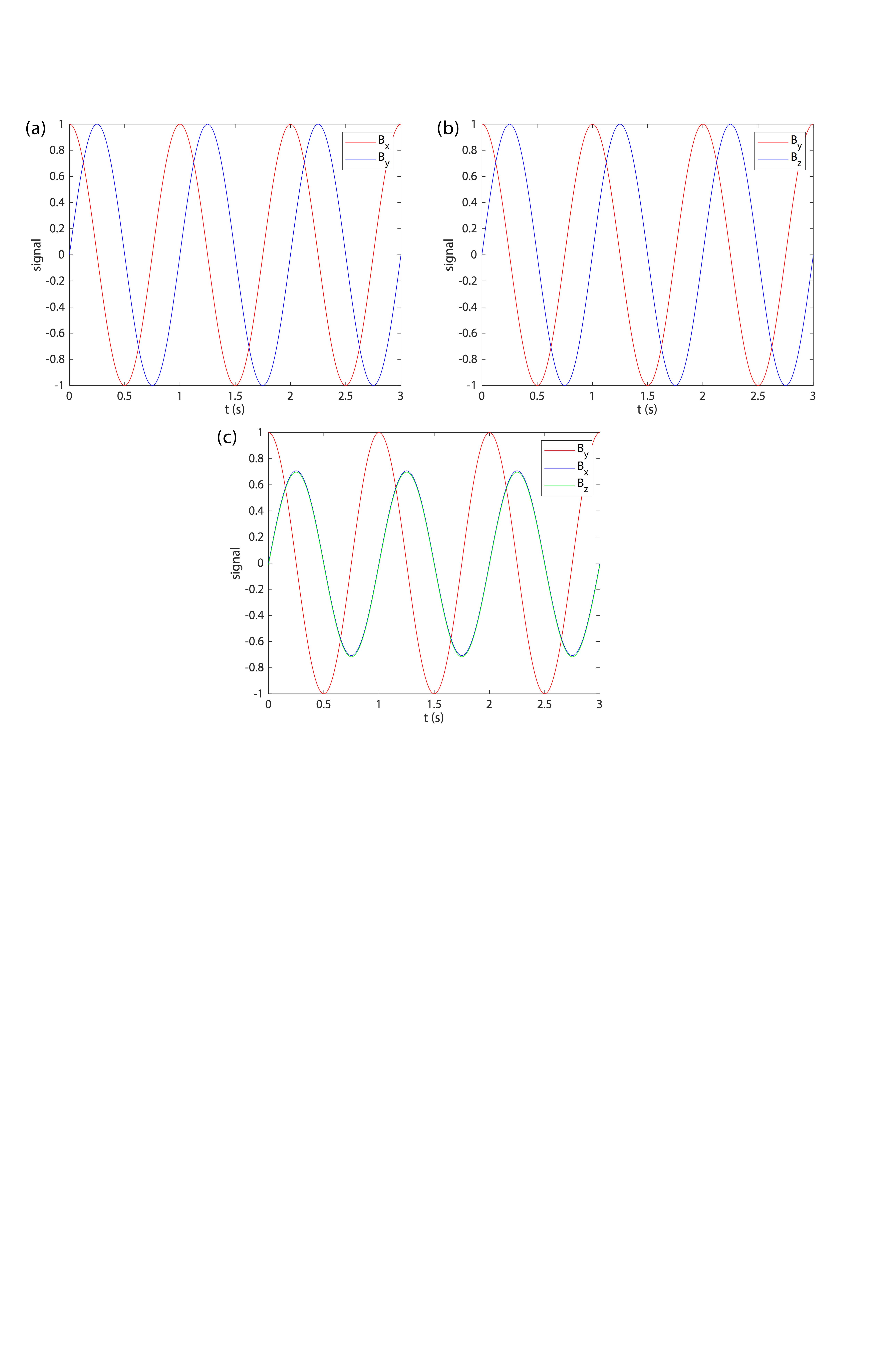
**

**Fig. S15. Driving magnetic field waveforms of the MagCap in various cases.** (a) Waveform of the driving magnetic field for rolling mode. (b) Waveform of the driving magnetic field for the turning mode. (c) Waveform of the driving magnetic field for MagCap rolling towards the 45-degree direction.

**Supplementary Tables**

**Table S1. Comparison of the proposed capsules with the ones previously reported in the literature.**

| **References** | **Actuated source** | **Built-in magnetic source** | **Targeted transport** | **Delivery/**  **sampling** | **Magnetic field or force**  **strength for driving** | **Size** (mm) | **Delivery/Sampling**  **Volume** (mm^3^) | **RDC** | **Overall or local deformation** | **Expandable functions** |
| --- | --- | --- | --- | --- | --- | --- | --- | --- | --- | --- |
| **[7]** | Motor | — | **×** | **√/×** | Motor power  (~250 mW) | D8.2*L24.5 | 342.6 | 0.13 | Local | - Mucus-clearing |
| **[8]** | MF & motor | Permanent magnet | **√** | **×/√** | — | D11.6*L32 | 350 | 0.1 | Local | - Camera |
| **[9]** | Spring | — | **×** | **√/×** | Mechanical force 9 N | D9*L15 | — | — | Local | - Oral delivery for macromolecules |
| **[10]** | Battery | — | **×** | **×/×** | — | D12*L27 | — | — | — | - Photodynamic therapy for Helicobacter pylori infection |
| **[11]** | MF^1^ | — | **×** | **×/×** | — | — | — | — | — | - Photodynamic therapy for cancer therapy |
| **[12]** | MF | Permanent magnet | **√** | **×/√** | Magnetic force 0.6 N | D17.8*L24.6 | — | — | Overall | - Fine-Needle Biopsy |
| **[13]** | MF  & Ultrasound | Magnetic core | **√** | **√/×** | 7 T | D5 | — | — | — | - MRI image |
| **[14]** | MF | Permanent magnet | **√** | **√/×** | 150 mT | D16*L32.5 | 800 | 0.12 | Overall | — |
| **[15]** | MF | Permanent magnet | **×** | **√/×** | 70 mT | D20*L40 | 800 | 0.06 | Overall | — |
| **[16]** | MF | Permanent magnet | **√** | **×/√** | 44.4 mT | D13*L32 | 5 | 0.001 | Local | - Camera - Biopsy |
| **[17]** | MF | Permanent magnet | **√** | **×/√** | 0.55 N | D15*L32 | 0.35 | 6.2×10^-5^ | Overall | - Biopsy |
| **[18]** | MF | Soft magnet | **√** | **√/×** | 375 mT | D12*L33 | 780 | 0.24 | Overall | — |
| **[19]** | MF | Permanent magnet | **√** | **√/×** | — | D14*L28.9 | 2045 | 0.46 | Local | - Multi-drug delivery |
| **[20]** | MF | Permanent magnet | **×** | **×/√** | 6 mT | D12.4*L26 | 1500 | 0.48 | Overall | — |
| **[21]** | MF | Permanent magnet | **×** | **×/√** | 15 mT | D8*L11 | 42 | 0.07 | Overall | — |
| **[22]** | MF | Permanent magnet | **-** | **×/√** | 103.5 mT | D9*L24 | 1 | 0.005 | Local | - Biopsy |
| **[23]** | MF | Magnetic composite  (Cobalt & NdFeB) | **-** | **-** | 295 mT | D0.5*L0.82 | 6.44×10^-3^**^#^** | 0.039**^#^** | Overall | — |
|  | MF | Magnetic composite | **√** | **√/√** | 100 mT | D0.5*L0.45 | 2.34×10^-^**^3#^** | 0.026**^#^** | Overall | — |
| **Our work** | MF | **Double-layer magnetic composites** | **√** | **√/√** | **30 mT** | **D5.8*L13**  **D8.4*L19.5** | **121.6**  **457.6** | **0.35**  **0.42** | **Local** | - Multi-drug delivery - Mucus-clearing - Light-assisted therapy - Thermal-assisted therapy |

**#:** It is not given directly in the original article but is obtained by our estimation.

**MF:** Magnetic Field; **1:** The magnetic field is only used as a wireless power supply.

**RDC**: Ratio of the volume of loaded drug or sampling to the total volume of the capsule.

**Overall deformation**: When the capsule fulfills the function of drug delivery or sampling, the overall structure of the capsule will change, such as separating into two parts or undergoing compression deformation.

**Local deformation**: When the capsule fulfills the function of drug delivery or sampling, the overall external structure and shape of the capsule remain unchanged, only the internal structure changes.

**[23]:** This type is technically capsule-shaped rather than a true capsule.

**Red font**: Indicating that the capsule size proposed in the aforementioned literature is larger than Size 00 capsules (8.53×23.3mm) or the required driving magnetic field is higher than 50mT.

**Table S2. Structural features of various capsules and their corresponding function.**

| **Capsule type** | **Dimension** | **Shell material** | **Structural features** | **Distinctive functions** |
| --- | --- | --- | --- | --- |
| **Cap1** | Refer to Fig. S1 | Transparent resin | Transparent shell | Capsule dynamics study |
| **Cap2** |  | MED610 photopolymer | Biocompatible shell | *In vivo* animal experiment |
| **Cap3** |  | Transparent resin | Dual-module | Multi-drug delivery |
| **Cap4** |  | MED610 photopolymer | Threaded shell | Mucus-clearing |
| **Cap5** |  | MED610 photopolymer | Having an additional LED induction light | Light-assisted therapy |
| **Cap6** |  | MED610 photopolymer | Having an additional elastomer with Fe_3_O_4_ nanoparticles | Thermal-assisted therapy |

**Table S3. Magnetic actuation systems used in different experimental scenarios.**

| **Figure number** | **Type of magnetic actuation systems** | **Magnetic field type** | **Magnetic field amplitude** | **Magnetic field frequency** |
| --- | --- | --- | --- | --- |
| **Fig. 2b-d** | Biaxial Helmholtz coil | Stable | 0-40 mT | - |
| **Fig. 3b** | Triaxial Helmholtz coil | Rotating (X-Y) | 10 mT | 0.5-20 Hz |
| **Fig. 3c, d** | Self-made coil | Sinusoidal | 30 mT | 10-30 Hz |
| **Fig. 4b, c** | **1.** Triaxial Helmholtz coil  **2.** self-made coil | Rotating (X-Y-Z) | **1.** 10 mT  2. 30 mT | **1.** 1Hz  2. 30 Hz |
| **Fig. 4d** | **1.** Permanent magnet (D50×L20 mm, N35)  **2.** Self-made coil | **1.** Rotating  **2.** Sinusoidal | **1.** -  **2.** 30 mT | **1.** <1 Hz  **2.** 30 Hz |
| **Fig. 5a-c** | **1.** A permanent magnet (D80×L80 mm, N35) on a 6-DOF arm  **2.** Self-made coil | **1.** Rotating  **2.** Sinusoidal | **1.** -  **2.** 30 mT | **1.** <1 Hz  **2.** 30 Hz |
| **Fig. 5e-i** | A poly-magnetic coil on a 6-DOF arm | Sinusoidal | 30 mT | 30 Hz |
| **Fig. 6** | **1.** Permanent magnet (D80×L80 mm, N35)  **2.** self-made coil | **1.** Rotating  **2.** Sinusoidal | **1.** -  **2.** 30 mT | **1.** <1 Hz  **2.** 30 Hz |
| **Fig. 7a, b** | Self-made coil | Sinusoidal | 15 and 25 mT | 30 Hz |
| **Fig. 7c, d** | Self-made coil | Sinusoidal | 15 mT and 30 mT | 5-10 Hz and 20-40 Hz |
| **Fig. 8a, b** | **1.** Permanent magnet (D50×L20 mm, N35)  **2.** Wireless powered transmitter coil | **1.** Rotating  **2.** Sinusoidal | **1.** -  **2.** - | **1.** <1 Hz  **2.** 35.7 kHz |
| **Fig. 8c, d** | **1.** Permanent magnet (D50×L20 mm, N35)  **2.** Self-made coil  **3.** Wireless heating coils | **1.** Rotating  **2.** Sinusoidal  **3.** Sinusoidal | **1.** -  **2.** 30 mT  **3.** - | **1.** <1 Hz  **2.** 30 Hz  **2.** 35.7 kHz |

**Table S4. Structural features of various coils. (**All units are in millimeters)

| Figure | Various coils | Components | Structural parameters | Value |
| --- | --- | --- | --- | --- |
| Fig. S5a | Biaxial Helmholtz coil | X-axis coils | Inner diameter | 128 |
|  |  |  | Outer diameter | 240 |
|  |  |  | Height | 150 |
|  |  | Y-axis coils | Inner diameter | 262 |
|  |  |  | Outer diameter | 400 |
|  |  |  | Height | 227 |
| Fig. S5b | Triaxial Helmholtz coil | X-axis coils | Inner diameter | 390 |
|  |  |  | Outer diameter | 500 |
|  |  |  | Height | 285 |
|  |  | Y-axis coils | Inner diameter | 260 |
|  |  |  | Outer diameter | 370 |
|  |  |  | Height | 210 |
|  |  | Z-axis coils | Inner diameter | 155 |
|  |  |  | Outer diameter | 242 |
|  |  |  | Height | 140 |
| Fig. S5e | Self-made coil | Z-axis coils | Inner diameter | 40 |
|  |  |  | Outer diameter | 200 |
|  |  |  | Height | 60 |

**References:**

1. Ju, Y. et al. Reconfigurable magnetic soft robots with multimodal locomotion. Nano Energy 87, 106169 (2021).

2. Sun, Y. et al. Hybrid-excited magneto-responsive soft actuators for grasping and manipulation of objects. Applied Materials Today 35, 101917 (2023).

3. Wen, H., Sun, Y., Liu, R., Li, L. & Cao, Q. Reprogrammable magnetization pattern and shape morphing of phase-change magnetic soft composites. Composites Communications 40, 101618 (2023).

4. Yao, J. et al. Adaptive Actuation of Magnetic Soft Robots Using Deep Reinforcement Learning. Advanced Intelligent Systems 5, 2200339 (2023).

5. Wang, L., Kim, Y., Guo, C. F. & Zhao, X. Hard-magnetic elastica. Journal of the Mechanics and Physics of Solids 142, 104045 (2020).

6. Wu, C., Xiang, Y., Qu, S., Song, Y. & Zheng, Q. Numerical study of millimeter-scale magnetorheological elastomer robot for undulatory swimming. J. Phys. D: Appl. Phys. 53, 235402 (2020).

7. Srinivasan, S. S. et al. RoboCap: Robotic mucus-clearing capsule for enhanced drug delivery in the gastrointestinal tract. Sci. Robot. 7, eabp9066 (2022).

8. Ding, Z. et al. Novel scheme for non-invasive gut bioinformation acquisition with a magnetically controlled sampling capsule endoscope. Gut 70, 2297–2306 (2021).

9. Abramson, A. et al. An ingestible self-orienting system for oral delivery of macromolecules. Science 363, 611–615 (2019).

10. Tortora, G. et al. An Ingestible Capsule for the Photodynamic Therapy of Helicobacter Pylori Infection. IEEE/ASME Trans. Mechatron. 21, 1935–1942 (2016).

11. Bansal, A., Yang, F., Xi, T., Zhang, Y. & Ho, J. S. In vivo wireless photonic photodynamic therapy. Proc. Natl. Acad. Sci. U.S.A. 115, 1469–1474 (2018).

12. Son, D., Gilbert, H. & Sitti, M. Magnetically Actuated Soft Capsule Endoscope for Fine-Needle Biopsy. Soft Robotics 7, 10–21 (2020).

13. Tiryaki, M. E., Doğangün, F., Dayan, C. B., Wrede, P. & Sitti, M. MRI-powered Magnetic Miniature Capsule Robot with HIFU-controlled On-demand Drug Delivery. in 2023 IEEE International Conference on Robotics and Automation (ICRA) 5420–5425 (IEEE, 2023). doi:10/gsjdg4.

14. Munoz, F., Alici, G., Zhou, H., Li, W. & Sitti, M. Analysis of Magnetic Interaction in Remotely Controlled Magnetic Devices and its Application to a Capsule Robot for Drug Delivery. IEEE/ASME Trans. Mechatron. 23, 298–310 (2018).

15. Yim, S., Goyal, K. & Sitti, M. Magnetically Actuated Soft Capsule With the Multimodal Drug Release Function. IEEE/ASME Trans. Mechatron. 18, 1413–1418 (2013).

16. Le, V. H. et al. Miniaturized biopsy module using gripper tool for active locomotive capsule endoscope. Mechatronics 44, 52–59 (2017).

17. Ye, D. et al. Design and Control of a Magnetically-Actuated Capsule Robot With Biopsy Function. IEEE Trans. Biomed. Eng. 69, 2905–2915 (2022).

18. Le, V. H. et al. A soft-magnet-based drug-delivery module for active locomotive intestinal capsule endoscopy using an electromagnetic actuation system. Sensors and Actuators A: Physical 243, 81–89 (2016).

19. Zheng, L., Guo, S. & Kawanishi, M. Magnetically Controlled Multifunctional Capsule Robot for Dual-Drug Delivery. IEEE Systems Journal 16, 6413–6424 (2022).

20. Nam, J., Lai, Y. P., Gauthier, L., Jang, G. & Diller, E. Resonance-based design of wireless magnetic capsule for effective sampling of microbiome in gastrointestinal tract. Sensors and Actuators A: Physical 342, 113654 (2022).

21. Shokrollahi, P. et al. Blindly Controlled Magnetically Actuated Capsule for Noninvasive Sampling of the Gastrointestinal Microbiome. IEEE/ASME Trans. Mechatron. 26, 2616–2628 (2021).

22. Simi, M., Gerboni, G., Menciassi, A. & Valdastri, P. Magnetic Torsion Spring Mechanism for a Wireless Biopsy Capsule. Journal of Medical Devices 7, 041009 (2013).

23. Zhang, J. et al. Voxelated three-dimensional miniature magnetic soft machines via multimaterial heterogeneous assembly. Sci. Robot. 6, eabf0112 (2021).
