# Supplementary material for: Magnetically driven capsules with multimodal response and multifunctionality for biomedical applications
Source: Nat Commun. 2024 Feb 29;15:1839. doi: 10.1038/s41467-024-46046-9 (PMC10904804; doi:10.1038/s41467-024-46046-9)
Supplement: Supplementary file 3 — Description of Additional Supplementary Files [file 41467_2024_46046_MOESM3_ESM.pdf]

Description of Additional Supplementary Files

Supplementary Movie 1. Sealing test for MagCaps with magnetic frames of different magnetic powder contents.

Supplementary Movie 2. Global motion performance of MagCaps under low-frequency magnetic field.

Supplementary Movie 3. Anchoring performance test of MagCaps under high-frequency magnetic field.

Supplementary Movie 4. Targeted transport and drug release/sampling processes in S- and U-shaped channels.

Supplementary Movie 5. Multimodal capsule locomotion in an anatomical model.

Supplementary Movie 6. Multi-target transport and drug delivery in an *ex vivo* pig stomach.

Supplementary Movie 7. Focused magnetic field performance test of a coil with an iron core at different distances in a water pipe model.

Supplementary Movie 8. Precise regional sampling using a coil with an iron core in a small intestine.

Supplementary Movie 9. *In vivo* validation of targeted transport and drug release in a rabbit stomach model.

Supplementary Movie 10. Sequential drug delivery process of a dual-module MagCap.

Supplementary Movie 11. Liquid mixing function of a MagCap with a threaded shell.

Supplementary Movie 12. Multi-functional demonstration of targeted transport and local illumination using a magneto-electric MagCap.

Supplementary Movie 13. Multi-functional demonstration of targeted transport, drug release, and local heating using a magneto-thermal MagCap.
