# Supplementary material for: Magnetically driven capsules with multimodal response and multifunctionality for biomedical applications
Source: Nat Commun. 2024 Feb 29;15:1839. doi: 10.1038/s41467-024-46046-9 (PMC10904804; doi:10.1038/s41467-024-46046-9)
Supplement: Supplementary file 17 — Reporting Summary [file 41467_2024_46046_MOESM17_ESM.pdf]

nature portfolio

Corresponding author(s): Quanliang Cao; Liang Li

Last updated by author(s): Quanliang Cao

Reporting Summary

Nature Portfolio wishes to improve the reproducibility of the work that we publish. This form provides structure for consistency and transparency in reporting. For further information on Nature Portfolio policies, see our Editorial Policies and the Editorial Policy Checklist.

Statistics

For all statistical analyses, confirm that the following items are present in the figure legend, table legend, main text, or Methods section.

n/a	Confirmed	
☐	☒	The exact sample size (*n*) for each experimental group/condition, given as a discrete number and unit of measurement
☐	☒	A statement on whether measurements were taken from distinct samples or whether the same sample was measured repeatedly
☒	☐	The statistical test(s) used AND whether they are one- or two-sided<br>*Only common tests should be described solely by name; describe more complex techniques in the Methods section.*
☒	☐	A description of all covariates tested
☒	☐	A description of any assumptions or corrections, such as tests of normality and adjustment for multiple comparisons
☐	☒	A full description of the statistical parameters including central tendency (e.g. means) or other basic estimates (e.g. regression coefficient) AND variation (e.g. standard deviation) or associated estimates of uncertainty (e.g. confidence intervals)
☒	☐	For null hypothesis testing, the test statistic (e.g. *F*, *t*, *r*) with confidence intervals, effect sizes, degrees of freedom and *P* value noted<br>*Give P values as exact values whenever suitable.*
☒	☐	For Bayesian analysis, information on the choice of priors and Markov chain Monte Carlo settings
☒	☐	For hierarchical and complex designs, identification of the appropriate level for tests and full reporting of outcomes
☒	☐	Estimates of effect sizes (e.g. Cohen's *d*, Pearson's *r*), indicating how they were calculated

Our web collection on statistics for biologists contains articles on many of the points above.

Software and code

Policy information about availability of computer code

Data collection	Bending angles of magnetic leaf in MagCaps were collected using Image J/Fiji (version of 2017) and Microsoft Excel 2019.<br>Distribution map of landing points in Fig.3c was collected using kinovea-0.9.5.
Data analysis	Magnetic field distribution map around the coil surface was analysed by the COMSOL Multiphysics 6.0.<br>Dynamic response characteristics of magnetic soft valves under an applied magnetic field was analysed by ABAQUS 2019.<br>The absorbance of the collected intestinal fluid was analysed by LabSolutions UV-Vis.

For manuscripts utilizing custom algorithms or software that are central to the research but not yet described in published literature, software must be made available to editors and reviewers. We strongly encourage code deposition in a community repository (e.g. GitHub). See the Nature Portfolio guidelines for submitting code & software for further information.

Data

Policy information about availability of data

All manuscripts must include a data availability statement. This statement should provide the following information, where applicable:

- Accession codes, unique identifiers, or web links for publicly available datasets
- A description of any restrictions on data availability
- For clinical datasets or third party data, please ensure that the statement adheres to our policy

All data supporting the findings of this study are available within the article and its supplementary files. Any additional requests for information can be directed to, and will be fulfilled by, the corresponding authors. Source data are provided with this paper.

Research involving human participants, their data, or biological material

Policy information about studies with human participants or human data. See also policy information about sex, gender (identity/presentation), and sexual orientation and race, ethnicity and racism.

Reporting on sex and gender	N/A.
Reporting on race, ethnicity, or other socially relevant groupings	N/A.
Population characteristics	N/A.
Recruitment	N/A.
Ethics oversight	N/A.

Note that full information on the approval of the study protocol must also be provided in the manuscript.

Field-specific reporting

Please select the one below that is the best fit for your research. If you are not sure, read the appropriate sections before making your selection.

☒ Life sciences ☐ Behavioural & social sciences ☐ Ecological, evolutionary & environmental sciences

For a reference copy of the document with all sections, see nature.com/documents/nr-reporting-summary-flat.pdf

Life sciences study design

All studies must disclose on these points even when the disclosure is negative.

Sample size	Four male New Zealand rabbits weighing 2.5-3 kg (purchased from WQJX-BIO Technology, Co., Ltd., China; License No. SCXK 2018-0020) were utilized for the magnetically driven capsule testing experiment under endoscope. In accordance with the principles of Animal experimental ethics and the Reduction component of the 3R principles, our animal experimental design was informed by prior studies on similar capsules (1. Yang, X. et al. An agglutinate magnetic spray transforms inanimate objects into millirobots for biomedical applications. Sci Robot 5, eabc8191 (2020).). Given the specific emphasis of our research on the magnetic-controlled release of capsules, with no intention to investigate the therapeutic effects on the organism post-drug release, and building upon the preliminary experiment of magnetic capsule efficacy in vivo, we conducted a repetition of four rabbit experiments to showcase the practical application effects of the product.
Data exclusions	No data were excluded from the analysis.
Replication	Four New Zealand rabbits of same gender and similar weight were selected for this study and reared in an identical environment for several days. Following a uniform fasting regimen, the rabbits underwent repeated magnetic-controlled capsule procedures under endoscopic surveillance after anesthesia. Drug release tests within the rabbit gastric cavity were conducted using a consistent dose of methylene blue, and experimental data were retained.
Randomization	A simple randomization approach was used to select rabbits co-housed together for sequential experimentation. In the animal experiment under endoscope, any one of the ten magnetic-controlled capsules of the same specifications was chosen using a simple randomization method.
Blinding	Most of our experimental results are presented as images. Blinding was not possible as experimental conditions were evident from the image data. Therefore, multiple group experiments were conducted to ensure the reliability of the results without excluding any data.

Reporting for specific materials, systems and methods

We require information from authors about some types of materials, experimental systems and methods used in many studies. Here, indicate whether each material, system or method listed is relevant to your study. If you are not sure if a list item applies to your research, read the appropriate section before selecting a response.

Materials & experimental systems

n/a	Involved in the study
☒	☐ Antibodies
☒	☐ Eukaryotic cell lines
☒	☐ Palaeontology and archaeology
☐	☒ Animals and other organisms
☒	☐ Clinical data
☒	☐ Dual use research of concern
☒	☐ Plants

Methods

n/a	Involved in the study
☒	☐ ChIP-seq
☒	☐ Flow cytometry
☒	☐ MRI-based neuroimaging

Animals and other research organisms

Policy information about studies involving animals; ARRIVE guidelines recommended for reporting animal research, and Sex and Gender in Research

Laboratory animals	Four male four-month-old New Zealand rabbits weighing 2.5-3 kg (purchased from WQJX-BIO Technology, Co., Ltd., China; License No. SCXK 2018-0020)
Wild animals	No wild animals were used in this study
Reporting on sex	The selection of animal genders (male rabbits) for experimentation in this study was carried out through a simple randomization process. This approach was adopted to prevent potential biases arising from gender differences in the experiment, particularly as the emphasis of this paper lies in the validation of capsule functionality.
Field-collected samples	No field-collected samples were used in this study.
Ethics oversight	All animal experiments were conducted in accordance with the protocol approved by the Laboratory Animal Ethics Committee of Tongji Medical College, Huazhong University of Science and Technology (Batch number: 3381).

Note that full information on the approval of the study protocol must also be provided in the manuscript.

Plants

Seed stocks	N/A.
Novel plant genotypes	N/A.
Authentication	N/A.
